# Clinical and genetic characteristics of patients with fatty acid oxidation disorders identified by newborn screening

**DOI:** 10.1186/s12887-018-1069-z

**Published:** 2018-03-08

**Authors:** Eungu Kang, Yoon-Myung Kim, Minji Kang, Sun-Hee Heo, Gu-Hwan Kim, In-Hee Choi, Jin-Ho Choi, Han-Wook Yoo, Beom Hee Lee

**Affiliations:** 1 Department of Pediatrics, Hanyang University Guri Hospital, Hanyang University College of Medicine, Guri, Korea; 2 Department of Pediatrics, Asan Medical Center Children’s Hospital, University of Ulsan College of Medicine, 88, Olympic-ro 43-Gil, Songpa-Gu, Seoul 05505 Korea; 3 Asan Insitute for Life Sciences, Asan Medical Center Children’s Hospital, 88, Olympic-ro 43-Gil, Songpa-Gu, Seoul 05505 Korea; 4 Medical Genetics Center, Asan Medical Center Children’s Hospital, University of Ulsan College of Medicine, 88, Olympic-ro 43-Gil, Songpa-Gu, Seoul 05505 Korea

**Keywords:** Fatty acid oxidation disorders, Newborn screening, Genotype-phenotype correlation, Treatment outcome

## Abstract

**Background:**

Fatty acid oxidation disorders (FAODs) include more than 15 distinct disorders with variable clinical manifestations. After the introduction of newborn screening using tandem mass spectrometry, early identification of FAODs became feasible. This study describes the clinical, biochemical and molecular characteristics of FAODs patients detected by newborn screening (NBS) compared with those of 9 patients with symptomatic presentations.

**Methods:**

Clinical and genetic features of FAODs patients diagnosed by NBS and by symptomatic presentations were reviewed.

**Results:**

Fourteen patients were diagnosed with FAODs by NBS at the age of 54.8 ± 4.8 days: 5 with very-long-chain acyl-CoA dehydrogenase (VLCAD) deficiency, 5 with medium chain acyl-CoA dehydrogenase (MCAD) deficiency, 1 with primary carnitine deficiency, 1 with carnitine palmitoyltransferase 1A (CPT1A) deficiency, 1 with long-chain 3-hydroxyacyl-CoA dehydrogenase or mitochondrial trifunctional protein (LCAHD/MTP) deficiency, and 1 with short chain acyl-CoA dehydrogenase (SCAD) deficiency. Three patients with VLCAD or LCHAD/MTP deficiency developed recurrent rhabdomyolysis or cardiomyopathy, and one patient died of cardiomyopathy. The other 10 patients remained neurodevelopmentally normal and asymptomatic during the follow-up. In 8 patients with symptomatic presentation, FAODs manifested as LCHAD/MTP deficiencies by recurrent rhabdomyolysis or cadiomyopathy (6 patients), and VLCAD deficiency by cardiomyopathy (1 patient), and CPT1A deficiency by hepatic failure (1 patient). Two patients with LCHAD/MTP deficiencies died due to severe cardiomyopathy in the neonatal period, and developmental disability was noted in CPT1A deficiency (1 patient).

**Conclusions:**

NBS helped to identify the broad spectrum of FAODs and introduce early intervention to improve the clinical outcome of each patient. However, severe clinical manifestations developed in some patients, indicating that careful, life-long observation is warranted in all FAODs patients.

## Background

Fatty acid oxidation (FAO) is a key metabolic pathway for maintaining energetic substrates used to maintain metabolic homeostasis. FAO is important for some high-energy-requiring organs and provides the main energy supply during prolonged fasting, febrile illness, cold exposure, or muscular exertion. The prime pathway for the degradation of fatty acids is mitochondrial fatty acid oxidation, which is composed of the uptake and activation of fatty acids, carnitine cycles, beta-oxidation cycle, and electron transfer [1].

More than 15 distinct disorders have been described as affecting FAO; These include glutaric aciduria type 2, primary carnitine deficiency and deficiencies of carnitine palmitoyltransferase 1A (CPT1A), carnitine acylcarnitine translocase (CACT), very long chain acyl-CoA dehydrogenase (VLCAD), long chain hydroxyacyl-CoA dehydrogenase or mitochondrial trifunctional protein (LCHAD/MTP), medium chain acyl-CoA dehydrogenase (MCAD), medium/short chain hydroxyacyl-CoA dehydrogenase (M/SCHAD), and short chain acyl-CoA dehydrogenase (SCAD) [2–4].

After the introduction of newborn screening by tandem mass spectrometry analysis of acylcarnitines, the detection of FAO disorders (FAODs) has increased. The estimated combined incidence of all FAODs is 1 in 9000, which is calculated from reports out of Australia, Germany, and the USA, but it seems to be much lower in Asian countries [5]. The estimated prevalence of long chain fatty acid oxidation disorders in Korea is 1 in 15,800, which is more frequently diagnosed following the introduction of tandem mass spectrometry newborn screening [6].

FAODs present with a heterogeneous clinical phenotype at various ages of onset, from neonate to adulthood. The most severe form manifests with hypertrophic cardiomyopathy, hepatic encephalopathy, or severe hypoketotic hypoglycemia in the neonatal period or infancy. The less-severe, later-onset myopathic form is characterized by exercise-induced myopathy and rhabdomyolysis [7, 8].

The diagnosis of FAODs are based on the measurement of abnormal acylcarnitines and confirmed by enzyme assay or molecular analysis. Early identification of FAODs became possible using expanded newborn screening using tandem mass spectrometry, which measures acylcarnitine levels in dried blood spots. Identification of FAODs in newborn screening is very important because intervention in the presymptomatic period helps improve patient prognosis [9].

In this respect, here we describe fourteen patients with diverse FAODs identified by newborn screening, and compare their clinical outcome and genetic characteristics with those of 8 patients diagnosed by symptomatic presentation. Our experience indicates the effectiveness of newborn screening for the early diagnosis of FAOD, especially in the presymptomatic period. However, careful observation and appropriate management is warranted to improve the clinical outcome of the affected patients.

## Methods

### Patients

A total of fourteen patients were diagnosed with FAODs by newborn screening between May 2002 and February 2016 at the department of Medical Genetics, Asan Medical Center Children’s Hospital, Seoul, Korea. Clinical features of these patients were compared with nine FAOD patients identified by symptomatic presentation, including rhabdomyolysis, cardiomyopathy, or developmental delay.

The Institutional Review Board at Asan Medical Center approved this study. Appropriate written informed consent was obtained from the parents of all participants.

#### Methods

Presenting manifestations, biochemical findings including plasma acylcarnitines, molecular analysis, and clinical course of each patient were reviewed retrospectively.

All patients diagnosed by newborn screening tests were referred to our department due to abnormal newborn screening results. Dried blood spot samples were obtained at the hospital where the patients were born and tandem mass spectrometry analysis was performed at commercial biochemical laboratories. Newborns with abnormal initial screening result were requested for repeated tandem mass spectrometry. If a second screening results were still exceeding the cut off value, molecular genetic analyses was performed and the diagnosis of FAOD was confirmed after receiving a positive confirmation test.

Acylcarnitine was measured by Liquid Chromatography-Tandem Mass Spectrometry, as previously described [6]. Genomic DNA was isolated from peripheral blood leukocytes. PCR was performed for all coding exons and exon–intron boundaries of *SLC22A5* for primary carnitine deficiency, *CPT1A* for CPT1A deficiency, *ACADVL* for VLCAD deficiency, *HADHA* and *HADHB* for LCHAD/MTP deficiencies, *ACADM* for MCAD deficiency, and *ACADS* for SCAD deficiency. Direct sequencing was performed on a ABI 3130 genetic analyzer (Applied Biosystems, Foster City, CA, USA) using a BigDye Terminator cycle sequencing kit (Applied Biosystems). In silico prediction analyses were performed for novel missense and splicing variants, using PolyPhen-2 (http://genetics.bwh.harvard.edu/pph2) and SIFT (http://sift.jcvi.org).

## Results

### Clinical characteristics of patients with FAODs identified by newborn screening

FAODs were identified in 14 patients by newborn screening: VLCAD deficiency (5 patients), MCAD deficiency (5 patients), primary carnitine deficiency (1 patient), CPT1A deficiency (1 patient), LCHAD/MTP deficiencies (1 patient), and SCAD deficiency (1 patient). Newborn screening using tandem mass spectrometry was performed at 3.2 ± 0.2 days after birth and the diagnosis of FAOD was confirmed by molecular genetic analyses at the mean age of 54.7 ± 40.8 days (range: 16–153 days). Plasma C14:1 and C14:2 were elevated in VLCAD deficiency, C14OH, C16OH and C18:1OH levels in LCHAD/MTP deficiencies, C6 and C8 in MCAD deficiency, C2 and C4 in SCAD deficiency, and C0/(C16 + C18) in CPT1A deficiency. C0 level was markedly decreased in primary carnitine deficiency.

### Molecular characteristics of patients with FAODs identified by newborn screening

Germline mutations of the gene responsible for each FAOD were identified in 90% of alleles (9 out of 10 alleles) in VLCAD deficiency, 90% (9 out of 10 alleles) in MCAD deficiency, 100% (2 out of 2 alleles) in LCHAD/MTP, primary carnitine, CPT1A, and SCAD deficiencies (Table 1). In VLCAD deficiency, 5 out of 7 mutations were novel mutations, c.[104_105ins10] (p.[Pro35fs*27]), c.[104_105ins5] (p.[Pro35fs*25]), c.[103_112dup] (p.[Arg38Profs*26]), c.[996_997ins(T)] (p.[Ala333Cysfs*26]), and c.[552C > G] (p.[Ile184Met])). Two novel mutations, c.[748G > T] (p.[Val250Leu]) and c.[1015C > T] (p.[Arg339Ter]), were detected in CPT1A deficiency. All other mutations were previously reported (Table 1) [10–20].

**Table 1:** Clinical, biochemical, and genetic characteristics of patients with fatty acid oxidation disorders diagnosed by newborn screening

No	Age at diagnosis	Age at last follow-up	Phenotype	Acylcarnitine		Gene	Allele 1	Allele 2
				Sample	Elevated acylcarnitine (value)			
Very long chain acyl-CoA dehydrogenase deficiency								
1	39 days	3.7 years	recurrent rhabdomyolysis and hypertrophic cardiomyopathy after 7 months old	DBS	C14 (2.504 μM; ref., 0.006–0.166), C14:1 (1.097 μM; ref., 0.006–0.166)	*ACADVL*	c.[104_105ins10] (p.[P35fs*27]) ^a^	c.[104_105ins5] (p.[P35fs*25]) ^a^
2	33 days	5.8 years	recurrent rhabdomyolysis after 11 months	DBS	C14:1 (n.a.), C14 (n.a.), C14:2 (n.a.)	*ACADVL*	c.[1349G > A] (p.[R450H])	c.[1349G > A] (p.[R450H])
3	25 days	10 months	hypertrophic cardiomyopathy	DBS	C14:2 (0.581 μM; ref., 0.006–0.166), C14:1 (1.391 μM; ref., 0.034–0.599)	*ACADVL*	c.[103_112dup] (p.[R38P*26]) ^a^	c.[1532G > A] (p.[R511Q])
4	49 days	3.3 years	1 episode of rhabdomyolysis	DBS	C14:1 (6.62 μM; ref., < 0.85)	*ACADVL*	c.[996_997ins(T)] (p.[A333C*26]) ^a^	c.[552C > G] (p.[I184M]) ^a^
5	48 days	2.0 years	asymptomatic	Plasma	C14 (0.184 μmol/L; ref., < 0.15), C14:2 (0.215 μmol/L; ref., < 0.13)	*ACADVL*	c.[1349G > A] (p.[R450H])	?
Medium chain acyl-CoA dehydrogenase deficiency								
6	16 days	4.5 years	asymptomatic	DBS	C8 (0.68 μM; ref., < 0.31)	*ACADM*	c.[617G > A] (p.[R206H])	c.[1189 T > A] (p.[Y397N])
7	36 days	3.5 years	asymptomatic	DBS	C6 (n.a.), C8 (n.a.), C10:1 (n.a.), C10 (n.a.)	*ACADM*	c.[1085G > A] (p.[G362E])	c.[1189 T > A] (p.[Y397N])
8	51 days	6.9 years	asymptomatic	DBS	C6 (0.46 μM; ref., < 0.22), C8 (1.66 μM; ref., < 0.35)	*ACADM*	c.[449_452del] (p.[Y150Rfs*4])	c.[1189 T > A] (p.[Y397N])
9	56 days	1.4 years	asymptomatic	DBS	C8 (2.98 μM; ref., < 0.37), C10:1 (0.58 μM; ref., < 0.40)	*ACADM*	c.[449_452del] (p.[Y150Rfs*4])	c.[1085G > A] (p.[G362E])
10	153 days	1.4 years	asymptomatic	Plasma	C6 (0.868 μmol/L; ref., < 0.18), C8 (5.067 μmol/L; ref., < 0.27), C10:1 (1.387 μmol/L; ref., < 0.46)	*ACADM*	c.[1189 T > A] (p.[Y397N])	?
Primary carnitine deficiency								
11	53 days	3.2 years	mild CK elevation, normal development	Plasma	C0 (4.1 μmol/L; ref., 12–46), Total carnitine (6.1 μmol/L; ref., 19–59)	*SLC22A5*	c.[396G > A] (p.[W132*])	c.[1400C > G] (p.[S467C])
Carnitine palmitoyltransferase 1A deficiency								
12	41 days	5 months	normal development	Plasma	C0 (80.839 μmol/L; ref., < 62.10), C0/(C16 + C18) (123.5)	*CPT1A*	c.[748G > T] (p.V250 L) ^a^	c.[1015C > T] (p.[R399*]) ^a^
Long chain hydroxyacyl-CoA dehydrogenase/mitochondrial trifunctional protein deficiencies								
13	26 days		Family history of sibling who died of lactic acidemia during the neonatal period. Died at age 49	DBS	C16OH (n.a.), C16OH/C16 (n.a.), C18:1OH (n.a.), C14 (n.a.), C14OH (n.a.)	*HADHA*	c.[1689 + 2 T > G] (deletion of exon 16)	c.[1689 + 2 T > G] (deletion of exon 16)
Short chain acyl-CoA dehydrogenase deficiency								
14	141 days	5 months	asymptomatic	Plasma	C4 (4.51 μmol/L; ref., < 1.06)	*ACADS*	c.[164C > T] (p.[P55L])	c.[1041A > G] (p.[E344G])

^a^indicates novel mutations. *DBS* dried blood spot samples, *n.a*. not available

### Clinical outcomes of patients with FAODs identified by newborn screening

The mean age at last follow-up for the 14 patients was 2.5 ± 2.0 years (range: 49 days–6.5 years). All patients had been educated to avoid prolonged fasting. Medium chain triglyceride diets with long chain fat restriction were recommended in the 5 patients with VLCAD deficiency and 1 patient with LCHAD/MTP deficiency. L-carnitine was given to 5 patients with MCAD deficiency even though the carnitine levels were within normal range, 1 patient with primary carnitine deficiency, and 1 patient with CPT1A deficiency.

Significant clinical manifestations that required emergency management were noted in 4 patients with VLCAD deficiency or LCHAD/MTP deficiency: recurrent rhabdomyolysis (2 patients), hypertrophic cardiomyopathy (2 patients), or sudden infantile death (1 patient). The remaining 2 patients with VLCAD deficiency and all patients with MCAD deficiency, SCAD deficiency, primary carnitine deficiency, or CPT1A deficiency were free of a metabolic crisis during the follow-up period. Additionally, the 13 surviving patients showed normal development without any neurologic deficit during the follow-up period (Table 1).

### Molecular and clinical characteristics of patients with FAODs identified by symptomatic presentation

During the same study period, a total of 8 patients were diagnosed with FAODs by symptomatic presentation (Table 2).

**Table 2:** Clinical, biochemical, and genetic characteristics of patients with fatty acid oxidation disorders diagnosed by clinical signs and symptoms

No	Age at diagnosis	Age at last follow-up	Phenotype	Acylcarnitine		Gene	Allele 1	Allele 2
				Sample	Elevated acylcarnitine (value)			
Long chain hydroxyacyl-CoA dehydrogenase/mitochondrial trifunctional protein deficiencies								
1	2.7 years	11.3 years	Recurrent rhabdomyolysis, sensorimotor polyneuropathy, difficulty running and climbing stairs	DBS	C10OH (n.a.), C18OH (n.a.)	*HADHB*	c.[340A > G] (p. [N114D])	c.[739C > T] (p.[R247C])
2	2.1 years	11.9 years	Recurrent rhabdomyolysis, sensorimotor polyneuropathy, difficulty running, positive Gowers’ sign	DBS	C10 (n.a.), C12 (n.a.), C14:1 (n.a.), C14OH (n.a.), C16OH (n.a.), C18:1OH (n.a.)	*HADHB*	c.[340A > G] (p. [N114D])	c.[919A > G] (p.[N307D])
3	4.8 years	6.8 years	Recurrent rhabdomyolysis, sensorimotor polyneuropathy, difficulty running	DBS	C14OH (n.a.), C16OH (n.a.), C18OH (n.a.), C18:1OH (n.a.)	*HADHB*	c.[340A > G] (p. [N114D])	c.[1148C > T] (p.[S383 L])
4	10.6 years	23.3 years	Recurrent rhabdomyolysis, sensorimotor polyneuropathy, walk with assistance	DBS	C14OH (0.156 μM; ref., 0.003–0.87), C16OH (0.228 μM; ref., 0.003–0.083), C18OH (0.072 μM; ref., 0.003–0.055)	*HADHB*	c.[919A > G] (p.[N307D])	c.[1165A > G] (p.[N389D])
5	1 day	–	Severe cardiomyopathy at first day of life. Died of lactic acidosis at 4 days old	DBS	C16OH, (0.86 μM; ref., < 0.15), C18OH (0.33 μM; ref., < 0.1), C18:1OH (0.48 μM; ref., < 0.08), C14:1 (0.66 μM; ref., < 0.35), C14 (1.35 μM; ref., < 0.86), C16:1 (0.54 μM; ref., < 0.25)	*HADHA*	c.[1793_1974del] (p.[H598Rfs*33])	c.[1793_1974del] (p.[H598Rfs*33])
6	5 days	–	Presented with tachypnea and metabolic acidosis at 5 days old. Died at 9 days old due to cardiomyopathy	DBS	C14 (n.a.), C14OH (n.a.), C16OH (n.a.), C18OH (n.a.), C18:1OH (n.a.)	*HADHB*	c.[1136A > G] (p.[H379R])	c.[1211dup] (p.[G404 fs*2]) ^a^
Very long chain acyl-CoA dehydrogenase deficiency								
7	2 months	3.9 years	Hypertrophic cardiomyopathy, recurrent rhabdomyolysis	DBS	C14:1 (n.a.), C14 (n.a.)	*ACADVL*	c.[997_998ins(T)] (p.[A333*]) ^a^	c.[1770_1773del] (p.[S590*]) ^a^
Carnitine palmitoyltransferase 1A deficiency								
8	33 months	6.8 years	Recurrent hepatic failure, nephromegaly, hemolytic anemia, rhabomyolysis, developmental delay	Plasma	C0 (68.86 μmol/L; ref., < 62.10), C0/(C16 + C18) (1639)	*CPT1A*	c.[837_838insT] (p.[I279*])	c.[947G > A] (p.[R316Q])

^a^indicates novel mutations. *DBS* dried blood spot samples, *n.a*. not available

One patient was diagnosed with VLCAD deficiency due to hypertrophic cardiomyopathy and rhabdomyolysis at the age of 2 months. This patient had two novel frameshift mutations in the *ACADVL* gene (p.A333fs and p.S590 fs). During the follow-up period of 3.5 years, this patient suffered from recurrent rhabdomyolysis and cardiomyopathy. LCHAD/MTP deficiencies were detected by cardiomyopathy with severe lactic acidosis at 1–5 days after birth (2 patients) and recurrent rhabdomyolysis at the median age of 9–48 months (4 patients). *HAHDA* or *HAHDB* mutations were identified in all six patients, including one novel mutation, c.[1211dup] (p.[G404 fs*2]). During the median follow-up period of 5.3 years (range: 4 days–12.7 years), the two patients with cardiomyopathy died at ages of 4 days and 9 days. The remaining four patients experienced recurrent rhabdomyolysis and sensorimotor polyneuropathy. No patient developed pigmentary retinopathy during follow-up. No patient was identified with MCAD or primary carnitine deficiency by symptomatic presentation during the study period.

One patient with CPT1A deficiency was identified by recurrent hepatopathy, nephromegaly, rhabdomyolysis, and hemolytic anemia [21]. Plasma acylcarnitine analysis revealed elevated free carnitine and ratio of free carnitine to C16 + C18. Compound heterozygous pathogenic mutations were identified in the *CPT1A* gene. Recurrent hepatic failure and intellectual disability was shown during 6.6 years of follow-up. At the age of 7, the patient’s intelligence quotient (IQ) was less than 35 and Korean Childhood Autism Rating Scale (K-CARS) score was 44, suggesting severe intellectual disability and severe autism.

## Discussion

The current study described the clinical and genetic features of 14 patients with FAODs identified by newborn screening, compared to those of 8 patients diagnosed with FAODs based on their symptomatic presentations.

The results of our current study indicate some important findings. Newborn screening helped to identify the broad spectrum of FAODs compared to FAODs with symptomatic presentation. These findings were comparable to previous reports regarding the newborn screening program [8, 22]. In addition, the relative frequency of each FAOD was reflected; VLCAD, LCHAD/MTP, and MCAD deficiencies were common FAODs identified by newborn screening. Alternatively, other FAODs, including primary carnitine deficiency, CPT1A or SCAD deficiencies, were identified in a small number of patients. Of note, VLCAD or LCHAD/MTP deficiencies were the most common among the FAODs identified either by newborn screening or by symptomatic presentation, whereas all patients with another common FAOD, MCAD deficiency, were identified by newborn screening, as symptomatic presentation of MCAD deficiency is expected to be very rare.

The purpose of newborn screening is to identify patients with inborn metabolic disorders in their presymptomatic period and intervene to prevent a metabolic crisis and improve their clinical outcome. In our current study, a fair outcome was observed in the patients with MCAD, CPT1A or primary carnitine deficiency identified by newborn screening. However, clinical outcomes were not significantly different among patients with long-chain FAODs, including VLCAD deficiency and LCHAD/MTP deficiencies identified either by newborn screening or by symptomatic presentation; most long-chain FAOD patients developed recurrent rhabdomyolysis and hypertrophic cardiomyopathy regardless of presymptomatic management.

The difference in outcomes among patients with FAODs appears to be related to disease characteristics and pathogenic effect of the mutations in addition to the mode of identification. In VLCAD or LCHAD/MTP deficiencies, patients identified by newborn screening experience a broad spectrum of severity even though the majority of patients were asymptomatic at diagnosis; symptoms develop in some patients even before newborn screening results were available, or some patients may remain asymptomatic through the long-term follow-up period [23–25]. Most of our patients with VLCAD or LCHAD/MTP deficiencies (4 out of 6) experienced recurrent rhabdomyolysis or severe cardiomyopathy. These severe phenotypes are related to complete inactivating or null alleles [25–28]. This correlation became more meaningful when the phenotype-genotype correlation was evaluated in all patients with VLCAD or LCHAD/MTP deficiencies, irrespective of the mode of identification. Seven patients with severe types of mutations, such as frameshift, nonsense, or splicing mutations, experienced severe phenotypes. Particularly, early death from severe cardiomyopathy was noted in 3 patients with severe mutations in LCHAD/MTP deficiencies. On the other hand, patients with missense mutations only either remained asymptomatic or experienced milder phenotypes (Tables 1 and 2).

As a long-term complication, peripheral polyneuropathy developed in four patients with LCHAD/MTP deficiencies, which has been reported in up to 80% of cases. In addition, pigmentary retinopathy develops in up 15–30% of patients [29, 30]. The mechanism responsible for these complications is not fully understood, although accumulation of 3-hydroxy fatty acid intermediate may be responsible [8]. Because the follow-up period was short for the patients in our current report, observation for these long-term complications is necessary.

The benign clinical course of MCAD deficiency can also be explained in part by the mutation type; most of the mutations were missense and only a small proportion of severe mutations such as frame-shift were noted. However, a life-long follow-up evaluation is required for patients with MCAD deficiency, considering the development of late-onset metabolic episodes. Between 3 and 24 months of age, patients may experience hypoketotic hypoglycemia, vomiting, lethargy, encephalopathy during febrile illness, or prolonged fasting. Sudden unexplained death may be the first presentation of MCAD deficiency [31, 32]. Even some adult patients may suffer from rhabdomyolysis, hepatic failure, encephalopathy or cardiac arrest triggered by alcohol consumption, pregnancy, or prolonged fasting [9, 33].

SCAD deficiency has also been classified as a benign condition because most of the newborns with SCAD deficiency as identified by newborn screening do not develop a clinical phenotype without any medical intervention. There exist controversies whether SCAD deficiency is benign biochemical phenotype, a clinical disorder with incomplete penetrance, or a clinically relevant part of multi-factorial or a multi-genetic disorder [34, 35]. Previously, some patients reported as having severe developmental delay, dysmorphic features and epilepsy, which would have been attributed to unknown genetic defects rather than to SCAD deficiency.

The CPT1A deficiency patient identified by newborn screening harbor heterozygous of two novel mutations. The patient remained asymptomatic during follow-up period, which was relatively short considering that CPT1A deficiency patients usually present by the age of 2 years [36]. The major manifestation of CPT1A deficiency is hepatic encephalopathy followed by febrile illness or prolonged fasting [21]. Even for a patient with neonatal presymptomatic presentation, late-onset hepatic failure develops, requiring lifelong evaluation. There exists a possibility that the novel missense mutation may not be deleterious, yet longer follow-up is needed in our patient in case of developing the symptomatic manifestations.

## Conclusions

Newborn screening for FAODs revealed the relative frequency of each disease subtype and their general clinical characteristics. This screening helped to reduce the mortality and morbidity of each patient with FAODs, but their broad spectrum of disease severity was also encountered regardless the mode of diagnosis, which was explained in part by their respective genotype. Careful, life-long observation of patients with FAODs is required to improve clinical outcome.

## Abbreviations

CPT1A: Carnitine palmitoyltransferase 1A
FAO: Fatty acid oxidation
FAODs: Fatty acid oxidation disorders
LCAHD/MTP: Long-chain 3-hydroxyacyl-CoA dehydrogenase or mitochondrial trifunctional protein
M/SCHAD: Medium/short chain hydroxyacyl-CoA dehydrogenase
MCAD: Medium chain acyl-CoA dehydrogenase
NBS: Newborn screenings
SCAD: Short chain acyl-CoA dehydrogenase
VLCAD: Very long chain acyl-CoA dehydrogenase

## References

[1] Houten SM, Wanders RJ (2010). A general introduction to the biochemistry of mitochondrial fatty acid beta-oxidation. J Inherit Metab Dis.

[2] Wanders RJ, Vreken P, den Boer ME, Wijburg FA, van Gennip AH, IJlst L (1999). Disorders of mitochondrial fatty acyl-CoA beta-oxidation. J Inherit Metab Dis.

[3] Rinaldo P, Matern D, Bennett MJ (2002). Fatty acid oxidation disorders. Annu Rev Physiol.

[4] Gregersen N, Andresen BS, Pedersen CB, Olsen RK, Corydon TJ, Bross P (2008). Mitochondrial fatty acid oxidation defects--remaining challenges. J Inherit Metab Dis.

[5] Lindner M, Hoffmann GF, Matern D (2010). Newborn screening for disorders of fatty-acid oxidation: experience and recommendations from an expert meeting. J Inherit Metab Dis.

[6] Yoon HR, Lee KR, Kang S, Lee DH, Yoo HW, Min WK, Cho DH, Shin SM, Kim J, Song J (2005). Screening of newborns and high-risk group of children for inborn metabolic disorders using tandem mass spectrometry in South Korea: a three-year report. Clin Chim Acta.

[7] Gregersen N, Andresen BS, Corydon MJ, Corydon TJ, Olsen RK, Bolund L, Bross P (2001). Mutation analysis in mitochondrial fatty acid oxidation defects: exemplified by acyl-CoA dehydrogenase deficiencies, with special focus on genotype-phenotype relationship. Hum Mutat.

[8] Spiekerkoetter U (2010). Mitochondrial fatty acid oxidation disorders: clinical presentation of long-chain fatty acid oxidation defects before and after newborn screening. J Inherit Metab Dis.

[9] Schatz UA, Ensenauer R (2010). The clinical manifestation of MCAD deficiency: challenges towards adulthood in the screened population. J Inherit Metab Dis.

[10] Choi JH, Yoon HR, Kim GH, Park SJ, Shin YL, Yoo HW (2007). Identification of novel mutations of the HADHA and HADHB genes in patients with mitochondrial trifunctional protein deficiency. Int J Mol Med.

[11] Waddell L, Wiley V, Carpenter K, Bennetts B, Angel L, Andresen BS, Wilcken B (2006). Medium-chain acyl-CoA dehydrogenase deficiency: genotype-biochemical phenotype correlations. Mol Genet Metab.

[12] Purevsuren J, Kobayashi H, Hasegawa Y, Mushimoto Y, Li H, Fukuda S, Shigematsu Y, Fukao T, Yamaguchi S (2009). A novel molecular aspect of Japanese patients with medium-chain acyl-CoA dehydrogenase deficiency (MCADD): c.449-452delCTGA is a common mutation in Japanese patients with MCADD. Mol Genet Metab.

[13] Yokoi K, Ito T, Maeda Y, Nakajima Y, Ueta A, Nomura T, Koyama N, Kato I, Suzuki S, Kurono Y (2007). Acylcarnitine profiles during carnitine loading and fasting tests in a Japanese patient with medium-chain acyl-CoA dehydrogenase deficiency. Tohoku J Exp Med.

[14] Ensenauer R, Winters JL, Parton PA, Kronn DF, Kim JW, Matern D, Rinaldo P, Hahn SH (2005). Genotypic differences of MCAD deficiency in the Asian population: novel genotype and clinical symptoms preceding newborn screening notification. Genet Med.

[15] Jethva R, Ficicioglu C (2008). Clinical outcomes of infants with short-chain acyl-coenzyme a dehydrogenase deficiency (SCADD) detected by newborn screening. Mol Genet Metab.

[16] Pedersen CB, Kolvraa S, Kolvraa A, Stenbroen V, Kjeldsen M, Ensenauer R, Tein I, Matern D, Rinaldo P, Vianey-Saban C (2008). The ACADS gene variation spectrum in 114 patients with short-chain acyl-CoA dehydrogenase (SCAD) deficiency is dominated by missense variations leading to protein misfolding at the cellular level. Hum Genet.

[17] Smelt AH, Poorthuis BJ, Onkenhout W, Scholte HR, Andresen BS, van Duinen SG, Gregersen N, Wintzen AR (1998). Very long chain acyl-coenzyme a dehydrogenase deficiency with adult onset. Ann Neurol.

[18] Hoffmann L, Haussmann U, Mueller M, Spiekerkoetter U (2012). VLCAD enzyme activity determinations in newborns identified by screening: a valuable tool for risk assessment. J Inherit Metab Dis.

[19] Nezu J, Tamai I, Oku A, Ohashi R, Yabuuchi H, Hashimoto N, Nikaido H, Sai Y, Koizumi A, Shoji Y (1999). Primary systemic carnitine deficiency is caused by mutations in a gene encoding sodium ion-dependent carnitine transporter. Nat Genet.

[20] Koizumi A, Nozaki J, Ohura T, Kayo T, Wada Y, Nezu J, Ohashi R, Tamai I, Shoji Y, Takada G (1999). Genetic epidemiology of the carnitine transporter OCTN2 gene in a Japanese population and phenotypic characterization in Japanese pedigrees with primary systemic carnitine deficiency. Hum Mol Genet.

[21] Lee BH, Kim YM, Kim JH, Kim GH, Kim JM, Kim JH, Woo KH, Yang SH, Kim CJ, Choi IH (2015). Atypical manifestation of carnitine palmitoyltransferase 1A deficiency: hepatosplenomegaly and nephromegaly. J Pediatr Gastroenterol Nutr.

[22] Landau YE, Waisbren SE, Chan LM, Levy HL (2017). Long-term outcome of expanded newborn screening at Boston children's hospital: benefits and challenges in defining true disease. J Inherit Metab Dis.

[23] Karall D, Brunner-Krainz M, Kogelnig K, Konstantopoulou V, Maier EM, Moslinger D, Plecko B, Sperl W, Volkmar B, Scholl-Burgi S (2015). Clinical outcome, biochemical and therapeutic follow-up in 14 Austrian patients with long-chain 3-Hydroxy acyl CoA dehydrogenase deficiency (LCHADD). Orphanet J Rare Dis.

[24] Sykut-Cegielska J, Gradowska W, Piekutowska-Abramczuk D, Andresen BS, Olsen RK, Oltarzewski M, Pronicki M, Pajdowska M, Bogdanska A, Jablonska E (2011). Urgent metabolic service improves survival in long-chain 3-hydroxyacyl-CoA dehydrogenase (LCHAD) deficiency detected by symptomatic identification and pilot newborn screening. J Inherit Metab Dis.

[25] Pena LD, van Calcar SC, Hansen J, Edick MJ, Walsh Vockley C, Leslie N, Cameron C, Mohsen AW, Berry SA, Arnold GL (2016). Outcomes and genotype-phenotype correlations in 52 individuals with VLCAD deficiency diagnosed by NBS and enrolled in the IBEM-IS database. Mol Genet Metab.

[26] Spiekerkoetter U, Lindner M, Santer R, Grotzke M, Baumgartner MR, Boehles H, Das A, Haase C, Hennermann JB, Karall D (2009). Management and outcome in 75 individuals with long-chain fatty acid oxidation defects: results from a workshop. J Inherit Metab Dis.

[27] Spiekerkoetter U, Sun B, Khuchua Z, Bennett MJ, Strauss AW (2003). Molecular and phenotypic heterogeneity in mitochondrial trifunctional protein deficiency due to beta-subunit mutations. Hum Mutat.

[28] Boutron A, Acquaviva C, Vianey-Saban C, de Lonlay P, de Baulny HO, Guffon N, Dobbelaere D, Feillet F, Labarthe F, Lamireau D (2011). Comprehensive cDNA study and quantitative analysis of mutant HADHA and HADHB transcripts in a French cohort of 52 patients with mitochondrial trifunctional protein deficiency. Mol Genet Metab.

[29] den Boer ME, Dionisi-Vici C, Chakrapani A, van Thuijl AO, Wanders RJ, Wijburg FA (2003). Mitochondrial trifunctional protein deficiency: a severe fatty acid oxidation disorder with cardiac and neurologic involvement. J Pediatr.

[30] Spiekerkoetter U, Bennett MJ, Ben-Zeev B, Strauss AW, Tein I (2004). Peripheral neuropathy, episodic myoglobinuria, and respiratory failure in deficiency of the mitochondrial trifunctional protein. Muscle Nerve.

[31] Iafolla AK, Thompson RJ, Roe CR (1994). Medium-chain acyl-coenzyme a dehydrogenase deficiency: clinical course in 120 affected children. J Pediatr.

[32] Chace DH, DiPerna JC, Mitchell BL, Sgroi B, Hofman LF, Naylor EW (2001). Electrospray tandem mass spectrometry for analysis of acylcarnitines in dried postmortem blood specimens collected at autopsy from infants with unexplained cause of death. Clin Chem.

[33] Lang TF (2009). Adult presentations of medium-chain acyl-CoA dehydrogenase deficiency (MCADD). J Inherit Metab Dis.

[34] Jethva R, Bennett MJ, Vockley J (2008). Short-chain acyl-coenzyme a dehydrogenase deficiency. Mol Genet Metab.

[35] Nochi Z, Olsen RKJ, Gregersen N (2017). Short-chain acyl-CoA dehydrogenase deficiency: from gene to cell pathology and possible disease mechanisms. J Inherit Metab Dis.

[36] Baruteau J, Sachs P, Broue P, Brivet M, Abdoul H, Vianey-Saban C, Ogier de Baulny H (2013). Clinical and biological features at diagnosis in mitochondrial fatty acid beta-oxidation defects: a French pediatric study of 187 patients. J Inherit Metab Dis.

